# State of the Art in PEG-Based Heat Transfer Fluids and Their Suspensions with Nanoparticles

**DOI:** 10.3390/nano11010086

**Published:** 2021-01-03

**Authors:** Alina Adriana Minea

**Affiliations:** Faculty of Materials Science and Engineering, “Gheorghe Asachi” Technical University of Iasi, 700050 Iasi, Romania; aminea@tuiasi.ro

**Keywords:** PEG, nanoparticles, convective heat transfer, correlations, thermophysical properties

## Abstract

Research on nanoparticle enhanced fluids has increased rapidly over the last decade. Regardless of several unreliable reports, these new fluids have established performance in heat transfer. Lately, polyethylene glycol with nanoparticles has been demarcated as an innovative class of phase change materials with conceivable uses in the area of convective heat transfer. The amplified thermal conductivity of these nanoparticle enhanced phase change materials (PCMs) over the basic fluids (e.g., polyethylene glycol—PEG) is considered one of the driving factors for their improved performance in heat transfer. Most of the research, however, is centered on the thermal conductivity discussion and less on viscosity variation, while specific heat capacity seems to be fully ignored. This short review abridges most of the recent investigations on new PEG-based fluids and is dedicated especially to thermophysical properties of the chemicals, while a number of PEG-based nanofluids are compared in terms of base fluid and/or nanoparticle type and concentration. This review outlines the possibility of developing promising new heat transfer fluids. To conclude, this research is in its pioneering phase, and a large amount of experimental and numerical work is required in the coming years.

## 1. Introduction

Phase change materials (PCMs) have formed one of the most popular topics in research for the last 25 years. Despite the large body of existing research on phase change material properties and applications and the several published review papers, it is worth outlining the polyethylene glycol (PEG) family as one of the challenging classes of phase change materials with direct application in heat transfer enhancement.

One of the first comprehensive reviews on thermal storage systems and PCMs was accomplished by Zalba et al. [1] in 2003. The authors listed more than 150 materials used in research as PCMs, extracted from 230 references. This outstanding contribution is presented under three sections: materials, heat transfer, and applications. PCM applications can be classified as ice storage, building applications, conservation and transportation of temperature sensitive materials, water tanks vs. PCM tanks, and many others.

Sharma et al. [2] also conducted a review on thermal energy storage (TES) systems with phase change materials and their applications. The authors discussed energy storage methods, outlining mechanical, electrical, thermal, and thermochemical energy storage applications. They also discussed the sufficient properties needed for a specific PCM to be used in the design of a TES system and concluded that a good phase change material should have good thermophysical, kinetic, and chemical properties, as follows [2]:

- Thermal properties: good phase transition temperature, good heat transfer capabilities (i.e., especially high thermal conductivity, high specific heat capacity), and a high latent heat;
- Physical properties: good phase equilibrium, high values for density, and small vapor pressure;
- Kinetic properties: no conditions for supercooling and a good crystallization rate;
- Chemical properties: a very good chemical stability over time, lack of toxicity and absence of fire hazard (i.e., lack of flammability), a very good compatibility with other materials from the same system (for example, the construction materials, if applicable to buildings), lack of or low degradation after a number of freezing or melting processes;
- Preferable economic indicators, such as abundancy, large scale availability, and low costs.

**Figure 1:**
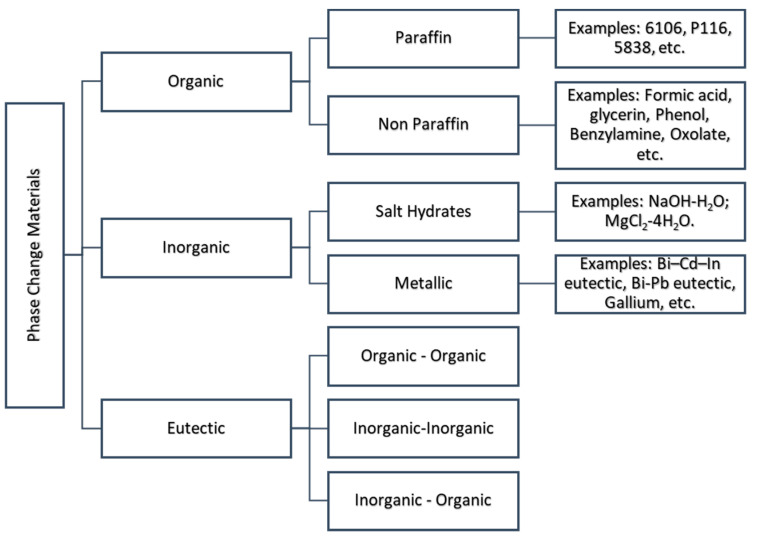
Phase change material (PCM) classification with several examples.

The classification of PCMs is illustrated in Figure 1. A complete list of all PCMs by category can be found in both Zalba et al. [1] and Sharma et al. [2].

**Figure 2:**
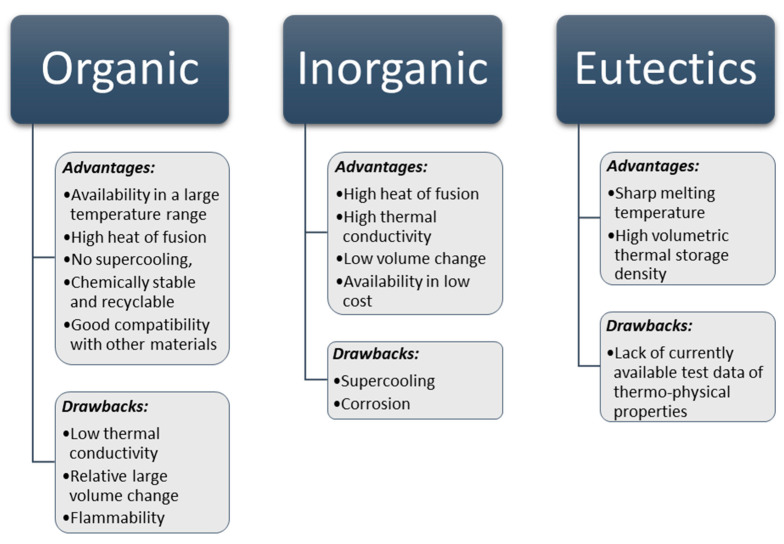
Advantages and drawbacks of PCMs.

The advantages and drawbacks of classes of PCMs are depicted in Figure 2.

This paper focuses on the polyethylene glycol (PEG) phase change material, though it is not our intention to deliver another review on PCMs, since many similar papers are already published in the open literature (see [1,2,3,4,5,6,7]). PEG has recently been developed as a green and low-cost catalyst as well as a solvent in several chemical transformations. It is a stable, recyclable, and biodegradable polymeric catalyst used successfully in organic synthesis, due to its possibility to be recycled numerous times, with a lack of noteworthy loss in its catalytic activity.

## 2. Polyethylene Glycol as a Phase Change Material

Polyethylene glycol (PEG), alongside paraffin and fatty acid, is an organic PCM, and it has a congruent phase change with a good nucleation rate (see Cabeza [7] for more details). Chemically, PEG is a polyether compound with countless uses, from industrial manufacturing to medicine. The structure of PEG is commonly expressed as H–(O–CH_2_–CH_2_)_n_–OH, and its application areas are outlined as follows:

- Chemical applications (as a lubricator, in biochemistry or biomembrane experimental studies, as a surfactant, as a calibration compound in mass spectrometry, etc.);
- Medicine (use as an excipient, for example);
- Biology (as a crowding agent, for protein crystallization)
- Commercial uses (in tattoos for monitoring diabetes, as an anti-foaming agent in several food and drink products, as a compound in skin creams, as a dispersant for toothpaste, etc.)
- Industrial applications (as an anti-foaming agent, in technical ceramics, as an insulator, etc.);
- Recreational applications.

The name PEG is usually followed by a number that signifies the average molecular weight of the compound. Table 1 depicts some basic data on studied forms of PEG, while Table 2 shows a summary of their thermophysical properties at ambient temperature (i.e., 25 °C) unless otherwise specified (see the viscosity values column).

## 3. Nanoparticle Enhanced PEG for Convective Heat Transfer Applications

PEG reveals high latent heat storage capacities at melting temperatures that can be adjusted by fluctuating the molecular mass of the polymer, as outlined in Table 1. In order to improve the thermal conductivity and the heat transfer behavior, nanoparticles are added to PEG, as in nanofluid or nanocomposite manufacturing.

This paper is dedicated to outlining and briefly discussing the thermophysical properties of state-of-the-art PEG-based heat transfer fluids obtained by suspending different kinds of nanoparticles in liquid polyethylene glycol. The suspension manufacturing follows the regular procedures applied to nanofluids, which include the mixing of the liquid with the nanoparticles, according to calculated quantities, depending on the final mass or volume fraction of nanoparticles in the fluid, and applying several sonication treatments (see [8] for example).

This section outlines the outcomes of studies on nanoparticle enhanced PEG in terms of its relevance as a new heat transfer fluid. In short, a new nanoparticle enhanced heat transfer fluid has to comply with several guidelines, such as high thermal conductivity and a moderate increase in viscosity. The discussion starts with thermal conductivity, which is by far the most examined thermophysical property. This section also contains an outline of density, viscosity, and specific heat capacity experimental results.

### 3.1. Thermal Conductivity

As stated, thermal conductivity of nanoparticle enhanced PEG is the most studied property, and a summary of experimental results identified from the open literature is presented in Table 3 [9,15,17,18,19,20,21,22,23,24].

As seen in Table 3, all the studies revealed an augmentation of thermal conductivity when nanoparticles were added to the base fluids. In terms of thermal conductivity variation with temperature, one can easily notice that most of the researchers found a decrease (see for example [9,17]), while some found that temperature has no major influence on experimental values (see for example [18,22]).

Marcos et al. [17] studied MWCNTs (Multi Wall Carbon Nanotubes) suspended in PEG 400 and compared the experimental data with several theoretical correlations. The researchers affirmed that all semi-empirical models underpredict the experimental values of thermal conductivity by at least 5.6%.

**Figure 3:**
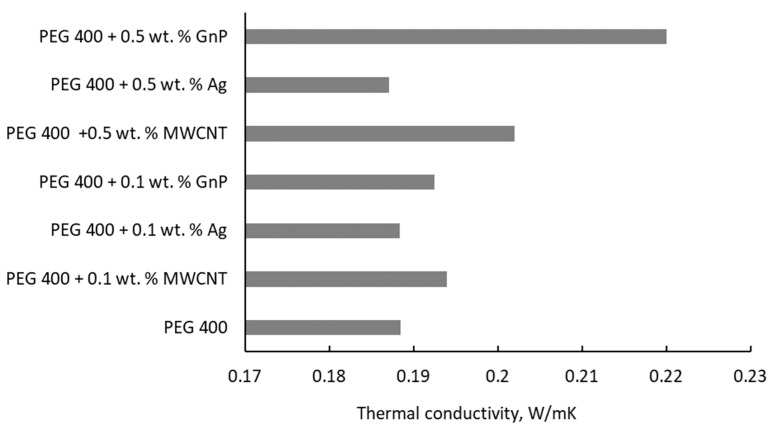
Thermal conductivity of several PEG-400-based fluids at 293 K [9,15,17].

Since most of the studies do not compare the experimental thermal conductivity of nanoparticle enhanced PEG, this paper gives an overview of the nanoparticle or base fluid influence. Figure 3 shows the nanoparticle type influence on thermal conductivity of PEG 400.

In Figure 3 it can clearly be seen that the influence of nanoparticle type and concentration is not consistent, since for 0.1 wt. % the augmentation is maximum for MWCNT addition, while for 0.5 wt. % the maximum is attained for graphene (GnP).

Unfortunately, no valid explanation on NP type influence was found in the literature, and a further comparison is not possible due to data scattering, as can be seen from Table 3.

To conclude, most of the experimental studies on PEG do not go deeper into the phenomenon of increasing the thermal conductivity. Overall, the thermal conductivity enhancement was found to be due to addition of highly conductive nanoparticles into the base fluids. Due to the lack of insight into experimental outcomes from different researchers, this author believes that the thermal conductivity augmentation mechanisms are similar to the ones noticed for other nanofluids. More exactly, these mechanisms are summarized in the open literature as: Brownian motion, surface charge, liquid–solid interface layer, and nanoparticle clustering. On the other hand, some influences have to be carefully studied, for example nanoparticle driven convection and convection prompted by electrophoresis or thermophoresis. Nevertheless, intense experimental studies on thermal conductivity are needed to completely reveal the processes that appear in these new nanoparticle enhanced fluids.

### 3.2. Density

Density experimental results are depicted in Table 4, and it can be noticed that very few studies have been conducted. Nevertheless, an initial conclusion is clear, pointing to two major conclusions:

1. Density decreases when temperature increases.
2. Density of the nanoparticle enhanced PEG slightly increases with nanoparticle concentration.

### 3.3. Viscosity

Viscosity results are also very few and are scattered in the literature, and this is mainly because this parameter was not monitored for most of these nanoparticle enhanced PEG due to their final application in real life cases.

Some comprehensive studies on PEG-based fluids were conducted by Marcos et al. [9,17,18], and their conclusion was that the viscosity increases with nanoparticle addition and decreases with temperature. All the base fluids were found to be Newtonian as while adding nanoparticles the flow behavior changed. This phenomenon is also observed for nanofluids.

Marcos et al. [17] found a shear-thinning behavior that was more pronounced with the growing MWCNT concentration. Similarly, a shear-thinning non-Newtonian behavior was also noticed by Yapici et al. [26] in their study of 1–10 wt. % TiO_2_ nanofluids based on PEG 200. More details are given in Table 5.

From the results depicted in Table 5 it can be seen that as the temperature increases, the viscosity decreases, and this is a normal phenomenon for most of the fluids. The phenomenon relies on the intermolecular attraction between the nanoparticles and their base fluids failing. Heating of most liquids leads to an increase of energy in the fluid. This intensification in energy increases the molecules’ random motion and fading of intermolecular forces holding the fluid molecules. This results in an decreased resistance of the fluid to shearing flow and thus a decrease in viscosity [28].

**Figure 4:**
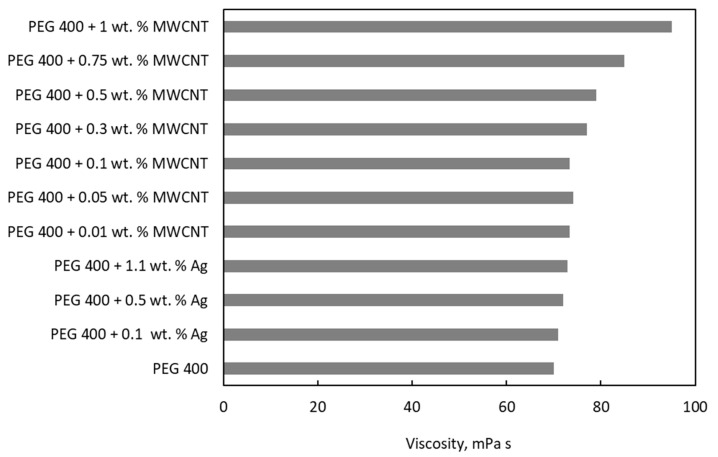
A comparison of viscosity values with PEG 400 as the base fluid.

The comparison of literature data in Figure 4 shows that the addition of Ag nanoparticles produces a slight increase in the viscosity of PEG, while 1% MWCNTs highly upsurge the PEG 400 viscosity.

**Figure 5:**
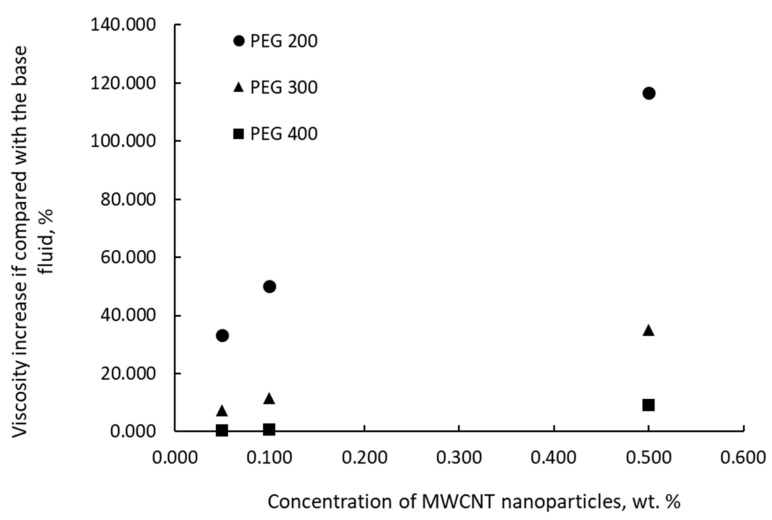
A comparison of adding MWCNTs to different PEG base fluids at 288.15 K.

Figure 5 depicts a comparison of experimental data on viscosity upsurge when MWCNT nanoparticles are added to three PEG base fluids (i.e., PEG 200, PEG 300, and PEG 400). Numbers show that if highly conductive nanoparticles are added to less viscous fluids (i.e., PEG 200 versus PEG 300 and PEG 400), the viscosity increase is higher, up to 110% for 0.5 wt. % MWCNTs in PEG 200.

To conclude, even various PEG base fluids were found to have a Newtonian flow behavior, nanoparticle addition modifies the flow into a non-Newtonian one, as all of available results demonstrated. Another observation is that adding nanoparticles increases the viscosity. This phenomenon is a normal one also encountered for regular nanofluids, and the explanation relies on the increase in shear rate particle–particle interactions that become weaker or are even broken down. Another explanation relies on the drag effect of individual nanoparticles (i.e., due to Brownian motion). Consequently, the global drag effect present in the medium is amplified, leading to an escalation in energy dissipation generating the augmentation in the nanofluid viscosity [28].

### 3.4. Specific Heat Capacity

Results for specific heat capacity are rather limited in the open literature and are briefly discussed, since no valid conclusion can be formed. Results of Marcos et al. [9,18] show that the specific heat capacity increases when nanoparticles are added and also increases with temperature.

Marcos et al. [9] performed experiments in the range 283.15–333.15 K with Ag–PEG 400 and found that adding nanoparticles to the phase change material has very low influence on specific heat capacity values.

Marcos et al. [18] measured specific heat capacity in the range 293–473 K and found an increase of 0.02–0.34% in specific heat capacity depending on the temperature and GnP weight concentration in PEG 400 phase change material. The maximum value was attained for 0.5% GnP at 293 K and the minimum for 0.05 wt. % GnP at 473 K.

## 4. Conclusions

This short review discusses polyethylene glycol, dispersions of nanoparticles in several polyethylene glycol fluids, and their possible use in thermal energy storage as nanoparticle enhanced phase change materials (NePCMs). According to studies identified in the open literature (see Marcos et al. [9] for example), the optimal preparation conditions consist of a sonication treatment with an ultrasonic homogenizer combined with mechanical stirring.

Results indicated that the nanoparticle addition certainly has a noticeable influence on the heat transfer behavior, since thermal conductivity increases along with specific heat capacity. Nevertheless, viscosity studies revealed a moderate increase that can overcome the disadvantages of using nanofluids.

To conclude, the studies on NePCMs are still in their pioneering phase, and experimental effort needs to be intensified, since no applicative studies were identified in the open literature. The PEG nanofluid stability in consecutive heating–cooling cycles and the possible hysteresis properties are also not discussed in the open literature. Thus, there is little critical evaluation of these nanoparticle-based PEG fluids, and more coordinated research is needed for a solid conclusion of the benefits and drawbacks of these new heat transfer fluids. No numerical studies were noticed from a careful search of the available databases.

Other nanoparticles (such as metallic nanoparticles, different carbon-based structures, and other kinds of nanoparticles) and other forms of PEG with different characteristics ought to be studied in future experiments for a complete analysis of the influence of both nanoparticle type and polymer chain dimension on PEG-based NePCMs.

## Figures and Tables

**Table 1:** Polyethylene glycol (PEG) type PCMs used for cool energy storage applications.

	Average Molecular Weight	Melting Point/Range, °C	Heat of Fusion, kJ/Kg	References
PEG 200	200	−50	not available	Gomez-Merino et al. [8]
PEG 400	400	5.8	105.3	Marcos et al. [9]
PEG 600	600	22.2	127.2	Demirbas [10] Ahmad et al. [11]
PEG 1000	1000	35–40	159	Azizi and Sadrameli [12]
PEG 1000 + PEG 600		23–26	150.5	Ismail and Castro [13]
PEG 2000	2000	35.93	172.13	Zhang et al. [14]
PEG 6000	6000	39.6	177.9	Tang et al. [15]
PEG 1500	1500	47.23	161.43	Kou et al. [16]
PEG 4000	4000	55.95	173.62	Kou et al. [16]
PEG 8000	8000	59.74	177.53	Kou et al. [16]
PEG 10000	10000	58.01	182.86	Kou et al. [16]
PEG 12000	12000	60.93	173.4	Kou et al. [16]
PEG 20000	20000	62.27	168.5	Kou et al. [16]

**Table 2:** Thermophysical properties of several forms of PEG, as identified in the literature.

	Thermal Conductivity, W/m K (at 298.15 K)	Viscosity, mPa s (at Different Temperatures)	Specific Heat Capacity (at 298.15 K)	Density, kg/m^3^ (at 298.15 K)	References
PEG 200	0.190	49.72 at 298.15 K	not available	1120.9	Gomez-Merino et al. [8]
PEG 400	0.152	70.44 at 298.15 K	not available	1003.8	Gomez-Merino et al. [8]
	0.184	73.4 at 303.15 K	2.350 kJ/kg K	1125.3	Marcos et al. [9]
PEG 600	not available	150 at 298.15 K	2.490 kJ/kg K	1128	Ahmad et al. [11]
PEG 1000	0.23	not available	2.142 kJ/kg K	1093	Azizi and Sadrameli [12]
PEG 1500	0.31	not available	2.473 kJ/kg K	1200	Kou et al. [16]
PEG 2000	0.31	not available	3.116 kJ/mol K	1210	
PEG 4000	0.33	not available	5.996 kJ/mol K	1200	
PEG 6000	0.2124 (0.34)	not available	not available (8.996 kJ/mol K)	1200	Tang et al. [15] (Kou et al. [16])
PEG 8000	0.33	not available	11.772 kJ/mol K	1270	Kou et al. [16]
PEG 10000	0.33	not available	14.455 kJ/mol K	1070	Kou et al. [16]
PEG 12000	0.32	not available	17.550 kJ/mol K	1200	Kou et al. [16]
PEG 20000	0.32	not available	28.180 kJ/mol K	1200	Kou et al. [16]

**Table 3:** Results for thermal conductivity.

Reference	Base Fluid	Nanoparticles	Concentration	Conditions	Observation
Marcos et al. [17]	PEG 400	MWCNT	0.01–1 wt. %	Temperature variation in the range 288.15–343.15 K	1. Thermal conductivity slightly decreases when temperature increases. 2. Thermal conductivity of the liquid inceases with nanoparticle concentration.
Marcos et al. [9]	PEG 400	GnP	0.05–0.5 wt. %	Temperature variation in the range 283–333 K	1. Thermal conductivity slightly decreases when temperature increases. 2. Thermal conductivity of the liquid inceases up to 23% with nanoparticle concentration.
Marcos et al. [18]	PEG 400	Ag	0.1–1.1 wt. %	Temperature variation in the range 283.15–333.15 K	1. Thermal conductivity remains almost constant when temperature increases. 2. Thermal conductivity of the liquid inceases with nanoparticle concentration.
Singh et al. [19]	PEG 1000	carbon powder	0.78 and 2.5 wt. %	Ambient temperature	Thermal conductivity of the liquid inceases up to 31% with nanoparticle concentration.
Yang et al. [20]	PEG 1000	GnP	up to 2.5 wt. %	Ambient temperature	Thermal conductivity of the liquid inceases up to 36% with nanoparticle concentration.
Liu et al. [21]	PEG 6000 + SiO_2_	carbon fiber	1–5 wt. %	Ambient temperature	Thermal conductivity inceases by 73% if compared to the base fluid and by 164% if compared to PEG 6000.
Qian et al. [22]	PEG 6000	SWCNT	2–10 wt. %	Temperature variation in the range 293.15–353.15 K	1. Thermal conductivity remains almost constant when temperature increases. 2. Thermal conductivity of the liquid inceases by 8.55 times at 10% nanoparticle concentration.
Tang et al. [15]	PEG 6000 + SiO_2_	Al_2_O_3_	3.3, 9.2, 12.6 wt. %	Ambient temperature	Compared to the pure PEG 6000 and PEG 6000/SiO_2_ data, the thermal conductivity of the composite PCM with Al_2_O_3_ inceases up to 46.5% and 20.8%, respectively.
Tang et al. [23]	PEG 6000 + SiO_2_	MWCNT	1–4 wt. %	Ambient temperature	Compared to the pure PEG 6000 and PEG 6000/SiO_2_ data, the thermal conductivity of the composite PCM with MWCNTs inceases up to 56% and 29%, respectively.
Cabaleiro et al. [24]	PEG 400	carbon black nano-diamonds graphite/diamond nanomixture	0.5 and 1 wt. %	Temperature variation in the range 288.15–318.15 K	1. Thermal conductivity remains almost constant when temperature increases. 2. Thermal conductivity of the liquid inceases up to 3.6% depending on nanoparticle type and concentration. The largest increase was for graphite/diamond nanomixture.

**Table 4:** Results for density.

Reference	Base Fluid	Nanoparticles	Concentration	Conditions	Observation
Marcos et al. [17]	PEG 400	MWCNT	0.01–1 wt. %	Temperature variation in the range 288.15–313.15 K	1. Density decreases when temperature increases 2. Density of the liquid increases up to 0.5% depending on nanoparticle concentration.
Marcos et al. [9]	PEG 400	GnP	0.05–0.5 wt. %	Temperature variation in the range 288.15–313.15 K	1. Density decreases when temperature increases 2. Density of the liquid increases up to 1.5% depending on nanoparticle concentration.
Marcos et al. [18]	PEG 400	Ag	0.1–1.1 wt. %	Temperature variation in the range 288.15–313.15 K	1. Density decreases when temperature increases 2. Density of the liquid increases up to 2.5% depending on nanoparticle concentration.
Cabaleiro et al. [24]	PEG 400	carbon black nano-diamonds graphite/diamond nanomixture	0.5% and 1 wt. %	Temperature variation in the range 288.15–313.15 K	1. Density decreases when temperature increases 2. Density of the liquid increases up to 40% depending on nanoparticle concentration and type.
Navidbakhsh and Majdan-Cegincara [25]	PEG 400, PEG 400 + PEG 2000, PEG 400 + PEG 6000	Fe_2_O_3_	0.1–31.8 vol. %	Temperature variation in the range 298.15–318.15 K	1. Density decreases when temperature increases 2. Density of the liquid increases up to 1.5% depending on nanoparticle concentration.

**Table 5:** Results for viscosity.

Reference	Base Fluid	Nanoparticles	Concentration	Conditions	Observation
Marcos et al. [17]	PEG 400	MWCNT	0.01–1 wt. %	Temperature variation in the range 288.15–343.15 K	Viscosity increases with nanoparticle addition up to 30% and decreases with temperature.
Marcos et al. [18]	PEG 400	Ag	0.1–1.1 wt. %	Temperature variation in the range 273.15–343.15 K	Viscosity increases with nanoparticle addition is very low. Viscosity decreases with temperature.
Cabaleiro et al. [24]	PEG 400	carbon black nano-diamonds graphite/diamond nanomixture	0.5 and 1 wt. %	Temperature variation in the range 288.15–318.15 K	1. Viscosity decreases when temperature increases 2. Viscosity of the suspensions increases up to 31.8% depending on nanoparticle type and concentration. The largest increase was for carbon black at 1 wt. %, and the minimum was 5% for 0.5% nanodiamond.
Yapici et al. [26]	PEG 200	TiO_2_	1–10 wt. %	Temperature variation in the range 263.15–313.15 K	PEG 200 has a Newtonian behaviour, and all new fluids are non-Newtonian. Viscosity increases with nanoparticle addition. An increasing shear thinning trend with temperature was noticed. Viscosity decreases with temperature.
Navidbakhsh and Majdan-Cegincara [25]	PEG 400, PEG 400 + PEG 2000, PEG 400 + PEG 6000	Fe_2_O_3_	0.1–31.8 vol. %	Temperature variation in the range 298.15–318.15 K	Several models existing in the literature were checked for compliance with experimental data. Newtonian behavior was observed for PEG 400 and shear thickening for both PEG 400 + PEG 2000 and PEG 400 + PEG 6000. A pseudoplastic behavior was noticed for all fluids with nanoparticles.
Marcos et al. [27]	PEG 200 PEG 300	MWCNT	0.025–0.7 wt. %	Temperature variation in the range 278.15–303.15 K	PEG 200 and PEG 300 have a Newtonian behaviour, and all new fluids are non-Newtonian. Viscosity increases with nanoparticle addition up to 105% and decreases with temperature.

## Data Availability

No new data were created in this study. Data sharing is not applicable to this article.
